# Cell-line screening and process development for a fusogenic oncolytic virus in small-scale suspension cultures

**DOI:** 10.1007/s00253-022-12027-5

**Published:** 2022-06-29

**Authors:** Sven Göbel, Fabian Kortum, Karim Jaén Chavez, Ingo Jordan, Volker Sandig, Udo Reichl, Jennifer Altomonte, Yvonne Genzel

**Affiliations:** 1 Max Planck Institute for Dynamics of Complex Technical Systems, Bioprocess Engineering, Sandtorstr. 1, 39106 Magdeburg, Germany; 2 Department of Internal Medicine II, Klinikum Rechts Der Isar, Technische Universität München, Munich, Germany; 3 ProBioGen AG, Herbert-Bayer-Str. 8, 13086 Berlin, Germany; 4 Chair for Bioprocess Engineering, Otto-Von-Guericke-University Magdeburg, Universitätsplatz 2, 39106 Magdeburg, Germany

**Keywords:** Cell line screening, Fusogenic oncolytic virus, Upstream processing, Cell culture-based production

## Abstract

**Abstract:**

Oncolytic viruses (OVs) represent a novel class of immunotherapeutics under development for the treatment of cancers. OVs that express a cognate or transgenic fusion protein is particularly promising as their enhanced intratumoral spread via syncytia formation can be a potent mechanism for tumor lysis and induction of antitumor immune responses. Rapid and efficient fusion of infected cells results in cell death before high titers are reached. Although this is an attractive safety feature, it also presents unique challenges for large-scale clinical-grade manufacture of OVs. Here we evaluate the use of four different suspension cell lines for the production of a novel fusogenic hybrid of vesicular stomatitis virus and Newcastle disease virus (rVSV-NDV). The candidate cell lines were screened for growth, metabolism, and virus productivity. Permissivity was evaluated based on extracellular infectious virus titers and cell-specific virus yields (CSVYs). For additional process optimizations, virus adaptation and multiplicity of infection (MOI) screenings were performed and confirmed in a 1 L bioreactor. BHK-21 and HEK293SF cells infected at concentrations of 2 × 10^6^ cells/mL were identified as promising candidates for rVSV-NDV production, leading to infectious titers of 3.0 × 10^8^ TCID_50_/mL and 7.5 × 10^7^ TCID_50_/mL, and CSVYs of 153 and 9, respectively. Compared to the AGE1.CR.pIX reference produced in adherent cultures, oncolytic potency was not affected by production in suspension cultures and possibly even increased in cultures of HEK293SF and AGE1.CR.pIX. Our study describes promising suspension cell-based processes for efficient large-scale manufacturing of rVSV-NDV.

**Key points:**

• *Cell contact-dependent oncolytic virus (OV) replicates in suspension cells.*

• *Oncolytic potency is not encompassed during suspension cultivation.*

• *Media composition, cell line, and MOI are critical process parameters for OV production.*

• *The designed process is scalable and shows great promise for manufacturing clinical-grade material.*

**Supplementary Information:**

The online version contains supplementary material available at 10.1007/s00253-022-12027-5.

## Introduction

OVs have emerged as a promising anti-cancer therapeutics due to their intrinsic ability to selectively infect and lyse tumor cells while inducing potent secondary immune responses (Krabbe and Altomonte 2018; Cook and Chauhan 2020). OVs expressing fusogenic glycoproteins are of particular interest as they lead to the formation of multinucleated giant cells (syncytia), facilitating intratumoral viral spread and tumor cell killing via multimodal responses that include the induction of adaptive immune responses directed against the tumor (Krabbe and Altomonte 2018). One promising OV in this immunotherapeutic class is rVSV-NDV, a recombinant hybrid virus constructed of a vesicular stomatitis virus (VSV) backbone and the surface glycoproteins of Newcastle disease virus (NDV) (Abdullahi et al. 2018). rVSV-NDV is a rapidly replicating and hyperfusogenic vector, providing the clinical benefits of each parental virus while eliminating the safety concerns associated with each. Proof-of-concept investigations indicate that rVSV-NDV is an effective and safe therapeutic for the treatment of hepatocellular carcinoma (Abdullahi et al. 2018) and murine melanoma, especially when administered in combination with adoptive T-cell transfer (Krabbe et al. 2021).

However, the low virus yields obtained from adherent cell cultures strongly limit the testable dose range in preclinical models. Despite screening of multiple adherent cell lines for rVSV-NDV production, the highest achievable titers remained relatively low (~ 10^6^ TCID_50_/mL in the culture harvest (Abdullahi et al. 2018) and a maximum of ~ 2–5 × 10^8^ TCID_50_/mL after purification and concentration). Because dose-limiting effects have not been observed using the virus concentrations, currently available processes with higher yields for attaining ≥ 10^8^ virions/injection are desirable. Furthermore, translation to the clinical application would be facilitated if efficient large-scale manufacturing processes free of animal-derived components can be developed.

Despite a favorable regulatory environment, current approaches to using adherent AGE1.CR.pIX cells for rVSV-NDV production pose major challenges for large-scale production (Abdullahi et al. 2018). This includes limited monitoring of pH value and dissolved oxygen partial pressure. In addition, restricted cell harvesting options and limited control of cultivation parameters such as feeding rates can complicate the scale-up of anchorage-dependent cells. In contrast, the use of suspension cells allows for an easier transfer from laboratory-bench systems to large-scale industrial bioreactors (Gallo-Ramírez et al. 2015). For the clinical-grade manufacturing of OV, the absence of animal-derived components in the cultivation media is highly desirable. In particular, the use of fetal calf serum can not only raise production and purification costs and cause higher batch-to-batch variations but also involves the risk of contamination with adventitious agents (Caron et al. 2018).

Even though wild-type VSV possesses a very broad tropism for various cell lines (Hastie et al. 2013), only a few publications reported the yield of VSV-based vectors from suspension cell culture (Elahi et al. 2019; Shen et al. 2019), and rVSV-NDV production in suspension cells has never been addressed before. The unique structure and fusogenic capacity of this hybrid virus likely cause altered tropism and replication kinetics and, consequently, demands the identification of suitable production cell lines. This may even include cell lines known to be tumorigenic or possessing abnormal karyology that are considered a risk in traditional vaccine manufacturing, due to differences in risk–benefit analysis (ICH 1998; Jordan and Sandig 2014). In addition, the high dose requirements of rVSV-NDV for further preclinical and first-in-human studies call for cell lines that achieve high cell-specific virus yields (CSVY). Furthermore, cell lines should be well-characterized, genetically stable, and demonstrated to be free of oncogenic viruses to facilitate subsequent clinical development (FDA 2010). Examples of safe and productive cell lines for vaccine and viral vector production include HEK293, AGE1.CR.pIX, EB66, Vero, and PER.C6 (Genzel 2015).

In the current study, we have evaluated the production of oncolytic rVSV-NDV-GFP (further referred to as VSV-NDV) in different suspension host cells under typical production conditions. Furthermore, virus adaptation and multiplicity of infection (MOI) screenings were performed to maximize virus titers. The findings were then applied to select cell lines that sustain high VSV-NDV titers and can be transferred to 1 L bioreactors for the establishment of a robust suspension cell-based process for oncolytic virus vector production. The latter was considered a critical requirement as oncolytic viruses are expected to enter mainstream clinical use. The screening and optimization steps outlined here can be easily adapted to other OV production platforms, particularly those involving fusogenic vectors, which present additional manufacturing challenges.

## Materials and methods

### Cell lines, media, and viral seed stock

A variety of different suspension cell lines (Supplement Table S1) were cultured and screened to identify a suitable cell substrate for the production of rVSV-NDV stocks. All cell lines had been adapted previously to anchorage-independent proliferation in suspension media. BHK-21 cells were maintained in Protein Expression Medium (Gibco, USA), supplemented with 8 mM L-glutamine (Sigma-Aldrich, USA) and 4 mM pyruvate (Sigma-Aldrich, USA) in the following PEM_s_. AGE1.CR and AGE1.CR.pIX cells were maintained in chemically defined CD-U7 medium (Xell, Germany) supplemented with 2 mM L-glutamine, 2 mM alanine (Sigma-Aldrich, USA), and 10 ng/mL recombinant insulin-growth factor (LONG-R^3^, Sigma-Aldrich, USA). MDCK cells were cultured in chemically defined Smif8 medium (protein- and peptide-free, Gibco, USA/K. Scharfenberg, FH Emden/Leer, Germany) supplemented with 4 mM L-glutamine and 4 mM pyruvate, or in chemically defined Xeno medium (Driving-M) (Shanghai BioEngine Sci-Tech, China) supplemented with 8 mM L-glutamine. HEK293SF cells were maintained in PEM_s_ medium, Dynamis medium, or FreeStyle™ 293 Expression Medium (Gibco, USA). Cell suspensions, except HEK293SF cells, were cultivated in baffled 125 mL flasks (50 mL *V*_w_, Corning, USA) at 185 rpm, 37 °C, and 5% CO_2_ in a Multitron Incubator Shaker (Infors AG, Switzerland) with a 5 cm orbit. HEK293SF cultures were agitated at 115 rpm, 37 °C, and 8% CO_2_.

The engineering, rescue, amplification, and purification of rVSV-NDV-GFP have been described previously (Abdullahi et al. 2018). The viral seed stock (6.58 × 10^7^ TCID_50_/mL) used in this study was produced in adherent AGE1.CR.pIX cells (ProBioGen AG, Germany) and stored at − 80 °C until use. For each subsequent experiment, a new aliquot of this stock was used to avoid potential loss of infectivity due to repeated freeze–thaw cycles.

### Oncolytic viral potency assay

As a measure for the oncolytic virus potency, adherent human hepatocellular carcinoma cells (Huh7) were infected with escalating serially diluted MOIs. Cell viability was measured using the CellTiter-Glo® Luminescent Cell Viability Assay (Promega Corp., Madison, USA). From the resulting dose–response curves, IC_50_ values were derived and interpreted as a readout for oncolytic potency. In brief, VSV-NDV-susceptible Huh7 cells were seeded in flat-bottom white-walled 96-well plates (# 655,088, Greiner Bio-One; Germany) at a density of 1 × 10^4^ cells/well using DMEM GlutaMAX media containing 10% fetal bovine serum and incubated overnight at 37 °C and 5% CO_2_. On the following day, serial half-log_10_ dilutions of the virus sample were prepared in medium to a minimum of one virion per well (MOI 10^−4^). For infection, the supernatant was removed and cells were infected with 100 µL of the prepared dilutions in quadruplicates, or left uninfected in fresh medium. A total of 48 h post-infection (hpi) the medium was replaced with 50 µL of fresh culture medium and 50 µL of the lytic assay substrate. Following the instructions in the kit manual, luminescence was recorded using a GloMax® Discover luminescence plate reader (Promega Corp., Madison, USA). After background subtraction of the medium, the reduction of cell viability was expressed relative to uninfected control cells and plotted against the log-MOI. Uninfected control cells were defined as 100% viable (top constraint = 1), whereas the bottom constraint was set to 0, representing 100% oncolysis.

### Small-scale infection screening

For experiments at conventional cell concentrations (conventional cell density, CCDs), shake flasks were inoculated at 6.0–8.0 × 10^5^ cells/mL and cultivated for 72 h to reach about 4.0–6.0 × 10^6^ cells/mL before infection. For experiments using higher cell concentrations, shake flasks were inoculated at 1.0 × 10^6^ cells/mL and cultivated for 72–96 h to reach about 1.0 × 10^7^ cells/mL. At the time of infection (TOI), the viable cell concentration was adjusted to CCD (2.0 × 10^6^ cells/mL) or higher cell concentrations (5.0 or 10.0 × 10^6^ cells/mL) by centrifuging the appropriate volume of cell suspension at 300 × *g* for 5 min and re-suspending in fresh medium containing virus. Host cells were infected at an MOI of 0.01. Additional shake flasks were supplemented with CaCl_2_ (0.3, 0.5, and 0.9 mM; Merck, Germany) to induce cell aggregation. Samples taken from the supernatant of infected cells were centrifuged for 5 min at 1100 × *g* to remove cellular debris, then aliquoted and stored at − 80 °C until further analysis.

For the screening of different MOIs, the same inoculation procedure was performed as described above for CCD experiments. MOIs ranging from 10^−1^ to 10^−5^ were tested. Samples taken from the supernatant of infected cells were centrifuged for 5 min at 1100 × *g* to remove cellular debris, then aliquoted and stored at − 80 °C until further analysis.

### Virus adaptation

VSV-NDV was adapted sequentially to the cell substrates following the inoculation procedure at CCD described previously. After the first infection, specified volumes of supernatant from each cell substrate were transferred to subsequent shake flasks with cells growing in the exponential growth phase at cell line-specific time points (Supplement Table S2). Subsequent passages were carried out at the same time point or earlier to select for fast-propagating viruses. Samples taken from cell culture supernatant were centrifuged for 5 min at 1100 × *g* to remove cellular debris, then aliquoted and stored at − 80 °C until further analysis.

### Infection in a small-scale bioreactor

Bioreactor cultures were performed in a DASGIP 1 L stirred tank bioreactor (STR) (Eppendorf AG, Germany) equipped with an InPro3100 pH sensor (Mettler Toledo, Switzerland), an InPro6800 polarographic dissolved oxygen sensor (Mettler Toledo, Switzerland), and a marine impeller. Additionally, an online capacitance probe connected to a controller (ArcView Controller 265, Hamilton, Switzerland) operating at a frequency range of 1 to 10 MHz was used to capture cell growth dynamics for future experiments. For aeration, a 3 sL/min air stream (21% O_2_) was supplied to the culture through a drilled hole L-sparger. DO was controlled at 50% air saturation by increasing the volumetric flow rate of the O_2_ content. The temperature was maintained at 37 °C using a heating blanket. For BHK-21 cells, the bioreactor was inoculated at a concentration of 0.5 × 10^6^ cells/mL, with pre-cultures expanded in 250 mL shake flasks and operated at a working volume (*V*_w_) of 350 mL, pH 7.2, and a stirring speed of 350 rpm. For HEK293SF cells, the bioreactor was inoculated at a concentration of 0.6 × 10^6^ cells/mL with pre-cultures expanded in 250 mL shake flasks and operated at *V*_w_ of 350 mL, pH 7.0, and stirring speed of 250 rpm. The pH was controlled at the respective set-point using 7.5% NaHCO_3_ or carbon dioxide. The same supplemented PEM_S_ medium was used for BHK-21 and HEK293SF batch cultures (Sect. 0). After the cells reached a concentration of 4.0 × 10^6^ cells/mL, 350 mL of fresh supplemented PEM_S_ medium containing virus was added, increasing the *V*_w_ to 700 mL. BHK-21 and HEK293SF cells were infected at an MOI of 10^−4^ and 10^−2^, respectively. Samples for metabolite analysis and virus titration were centrifuged at 1100 × *g* for 5 min to remove cell debris, aliquoted, and stored at − 80 °C until further analysis.

### Analytics

Percentage of viability and viable cell concentration were determined using an automatized ViCell™ XR cell viability analyzer (Coulter Beckman, USA). MDCK cells cultivated in Smith-8 medium were additionally incubated with 1 × trypsin for 10 min at 37 °C prior to measurement to break up aggregates. The metabolites, lactate, ammonium, glutamine, glutamate, and glucose were determined in single measurements with the BioProfile® 100 Plus (Nova Biomedical, USA). Virus-containing samples were inactivated at 80 °C for 3 min before metabolite measurements to allow easier handling. For titration of VSV-NDV, a modified version of the previously described TCID_50_ assay (Nikolay et al. 2020) was performed using adherent AGE1.CR.pIX cells (standard deviation of ± 0.3 log_10_ (TCID_50_/mL)). The CSVY was calculated as previously described by Gränicher et al. (2021), taking into account only the error of the TCID_50_ assay (− 50%/ + 100% on a linear scale).

### Flow cytometry

Flow cytometry was used to determine the percentage of VSV-NDV-infected cells at different time points post-infection. A total amount of 2.0 × 10^6^ cells was fixed using 4% paraformaldehyde for 30 min at 4 °C. Fixed cells were washed twice with FACS buffer [PBS, 2% (*w*/*v*) Glycin, 0.1% (*w*/*v*) BSA] (300 × *g*, 10 min, 4 °C) and re-suspended in 15 µL buffer. Analysis was performed using the ImageStream X Mark II (Amnis, EMD Millipore) by measurement of 10,000 single cells (debris and aggregates were excluded) at an excitation wavelength of 488 nm for detecting GFP + infected cells. Data analysis was carried out using the IDEAS software.

### Statistical analysis

Using a nonlinear regression model in GraphPad Prism (Version 8), IC_50_ values were calculated and compared between the data sets using the extra-sum of squares *F*-test.

## Results

### Screening for host cell selection

**Fig. 1:**
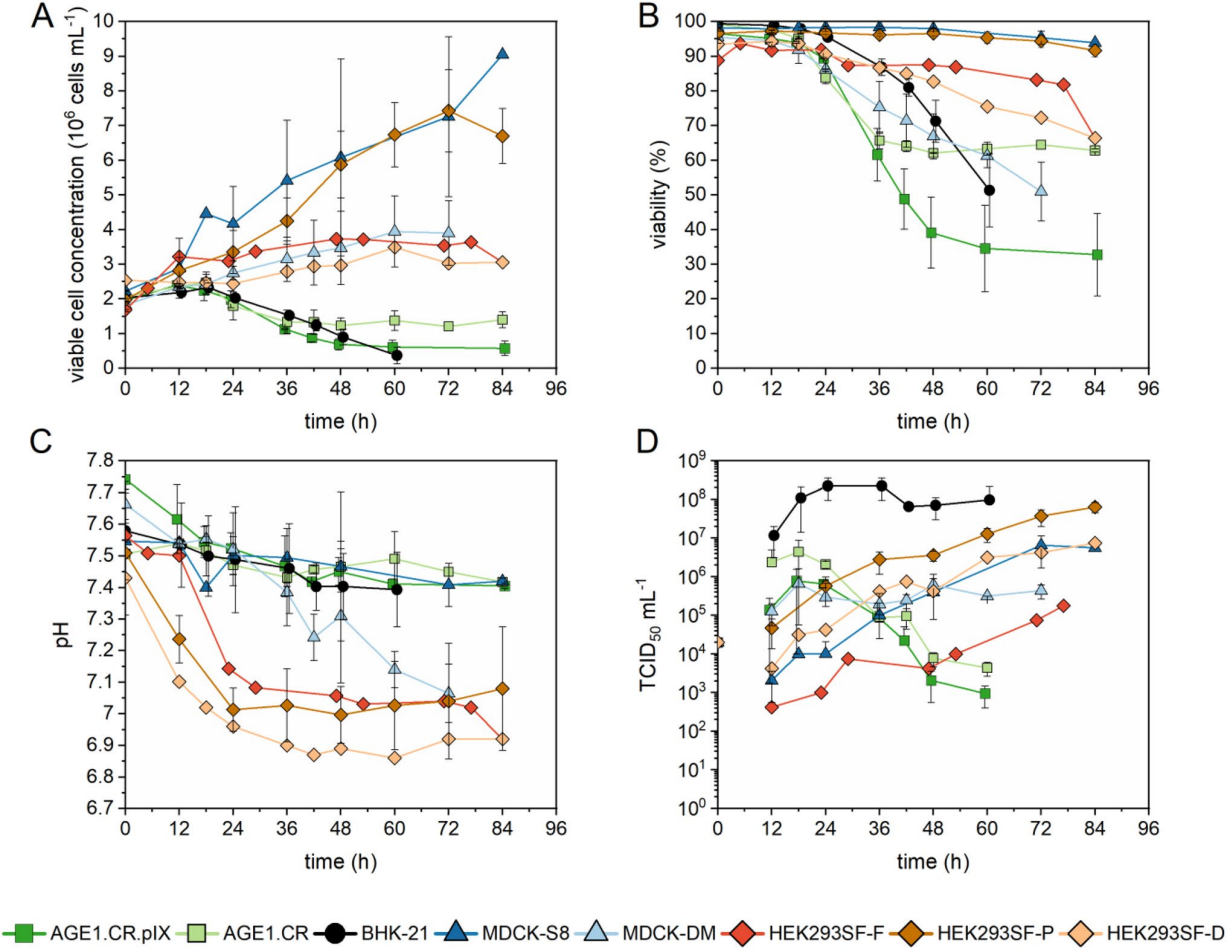
Growth characteristics of different cell lines after infection with VSV-NDV. Cell growth (**A**), viability (**B**), pH (**C**), and TCID_50_/mL (**D**) were compared between five cell lines. The medium was exchanged completely before infection. Cells were grown in baffled 125 mL shake flasks and infected at a viable cell concentration of 2 × 10.^6^ cells/mL with VSV-NDV at an MOI of 1E-2. The TCID_50_/mL at 0 hpi was calculated empirically, corresponding to an MOI of 1E-2 (only yellow symbol is visible, as the same MOI was used for all infections). Values (except HEK293SF-F and HEK293SF-D cells) are reported as the mean of a biological duplicate with two independent shake flasks. Error bars represent the standard deviation of duplicates

In order to identify cell lines that have the capacity to produce high titers of VSV-NDV, we first performed a screening of five suspension cell lines using conventional cell concentrations of 2 × 10^6^ cells/mL infected at an MOI of 0.01. For some of the cell lines, multiple media formulations were compared. As shown in Fig. 1, all cell lines tested could be infected with VSV-NDV but displayed different cell growth characteristics post-infection in shake flasks. MDCK cells growing in Smith-8 medium (MDCK-S8) and HEK293SF cells growing in PEM_s_ medium (HEK293SF-P) achieved the highest concentrations after infection (9.1 ± 1.2 × 10^6^ cells/mL and 7.4 ± 1.1 × 10^6^ cells/mL, respectively). They also displayed high viabilities (above 90%) until 84 hpi (Fig. 1A,B). BHK-21, AGE1.CR, and AGE1.CR.pIX cell concentrations stagnated at 2.4 ± 0.1 × 10^6^ cells/mL until 12 hpi and subsequently began to decrease, whereas HEK293SF cells in Freestyle medium (HEK293SF-F) and Dynamis medium (HEK293SF-D), as well as MDCK cells in Driving-M medium (MDCK-DM), slowly increased their cell concentration until the end of the cultivation period, reaching 3.0–3.9 ± 0.9 × 10^6^ cells/mL (Fig. 1A). The pH decreased during the infection period for all cell lines, ranging from 6.9 to 7.6 depending on the cell type and medium (Fig. 1C). Sharp declines in viability (up to 30%) starting at about 24 hpi were observed for AGE1.CR.pIX, AGE1.CR, and BHK-21 cells. Generally, a continuous growth phase after infection resulted in higher cell viability at the later stages of infection (Fig. 1B). The formation of syncytia, monitored by brightfield and fluorescence (GFP) microscopy, was not observed in any suspension cell line (data not shown). Next, we measured the number of infectious virus particles in the supernatant by TCID_50_ assay. All cell lines showed evidence of virus replication and release starting from 12 hpi. For HEK293SF-DM, HEK293SF-F, HEK293SF-P, MDCK-S8, and MDCK-DM cells, the VSV-NDV titer increased during the entire infection period to a maximum ranging from 10^5^ to 10^8^ TCID_50_/mL. Virus titers above 10^7^ TCID_50_/mL were only achieved using BHK-21 and HEK293-SF-P cells, but the virus replication dynamics differed. A strong correlation between maximum viable cell concentration and maximum TCID_50_/mL was observed for all cell lines (Fig. 1A,D).

Interestingly, the VSV-NDV titer produced by HEK293SF cells progressively increased over the entire cultivation period and could be enhanced over 2 logs by using different growth media. This was also true for MDCK cells, where cultivation in the Smith-8 medium resulted in 1 log higher titers compared to the Driving-M medium. After reaching the maximum titer, AGE1.CR.pIX and AGE1.CR cells showed a sharp decline in TCID_50_/mL with reductions up to 3 logs over 3 days (Fig. 1D). BHK-21 cells achieved the highest VSV-NDV titer (up to 2 ± 1 × 10^8^ TCID_50_/mL), with only a 0.5 log_10_ titer reduction over 3 days in this initial screening experiment. Although both AGE1.CR and AGE1.CR.pIX cells as well as BHK-21 cells exhibited a strong decline in cell concentration starting at 18 hpi, and the viral titer in BHK-21 cell cultures remained relatively stable. This may indicate the presence of a component in the PEM_s_ medium that could prevent virus degradation.

**Fig. 2:**
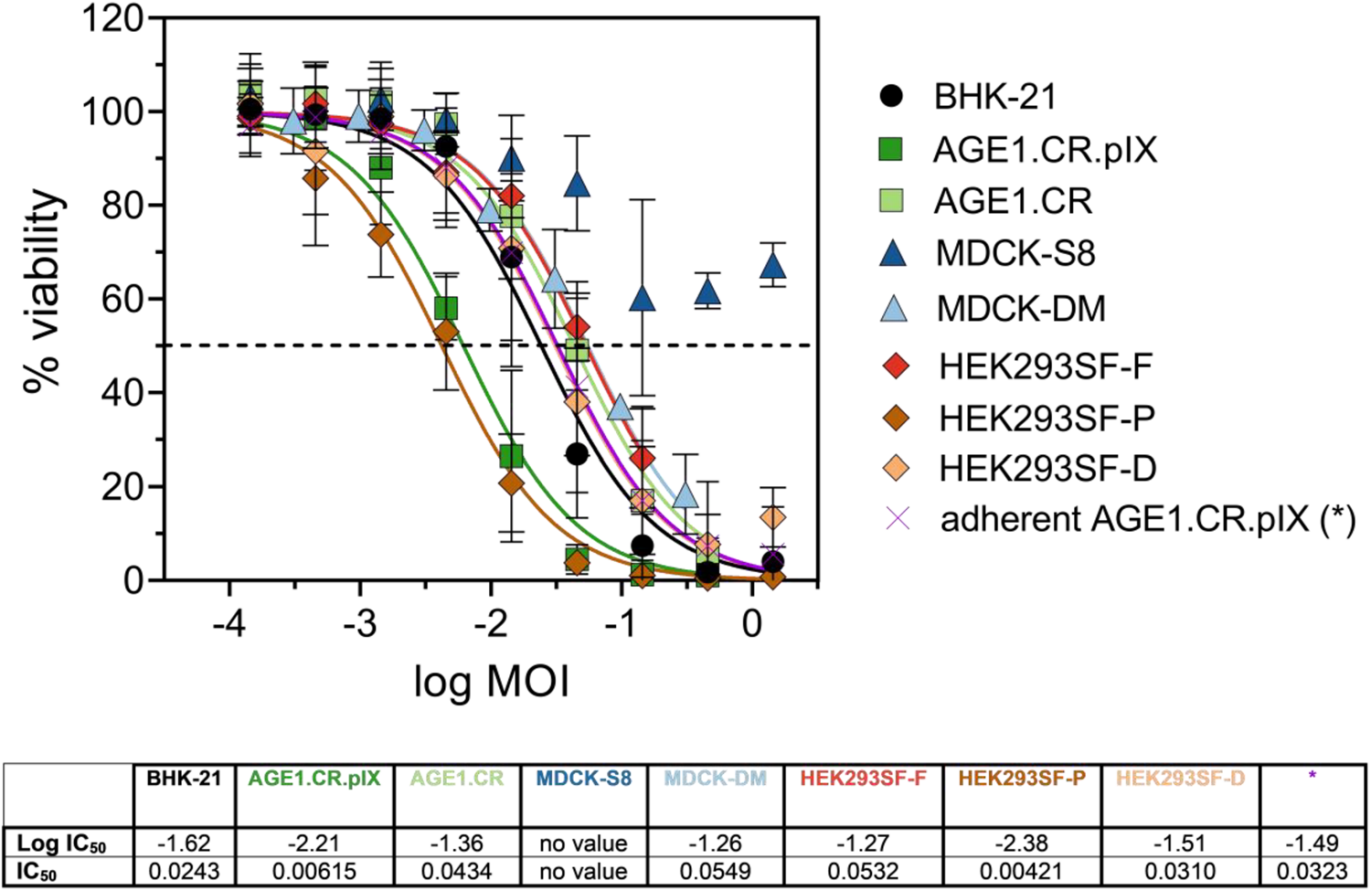
Oncolytic viral potency assay for VSV-NDV produced in suspension cells. Huh7 cancer cell viability was determined at 48 hpi with VSV-NDV-GFP generated in the respective suspension cell line and compared to virus generated in adherent AGE1.CR.pIX cells (*). The graph shows dose–response curves after non-linear regression analysis to calculate the individual IC_50_ and logIC_50_ values. Values are reported as the mean ± standard deviation of technical replicates, with *n* = 3 for suspension samples and *n* = 8 for adherent AGE1.CR.pIX

In order to rule out that the OV produced in suspension cultures did not alter its ability to efficiently kill cancer cells in vitro, an oncolytic viral potency assay was designed, similar to a previously described method (Almstätter et al. 2015). Peak titers from the individual suspension cultivations infected at MOI 0.01 were compared to the viral supernatant from adherent AGE1.CR.pIX cells infected at MOI 0.01 (peak titers, 24 hpi, Figure S1). Susceptible human hepatocellular carcinoma cells were infected at defined, serial MOI dilutions, and at 48 hpi, cell viability was determined and plotted as dose–response curves normalized to uninfected cells. IC_50_ values were derived and interpreted as a quantitative measure of VSV-NDV oncolytic potency (Fig. 2). For most cultivation conditions (BHK-21, HEK293SF-F, HEK293SF-D, MDCK-DM, and AGE1CR.pIX cells), oncolysis in Huh7 cells was only marginally affected and resulted in similar logIC_50_ values compared to virus generated in adherent AGE1.CR.pIX cells. Only virus sampled from MDCK cells infected in Smif8 medium was not able to induce more than 50% oncolysis, and hence, no IC_50_ value is reported. Of note, VSV-NDV generated in suspension AGE1.CR.pIX cells or HEK293SF-P cells cultivated in PEM_s_ medium showed a clearly superior oncolytic potency, defined by almost 1 log lower IC_50_ values.

### Effect of cell concentration and CaCl_2_ supplementation on VSV-NDV production

Since rVSV-NDV is a fusogenic oncolytic virus, which spreads through the formation of syncytia (Abdullahi et al. 2018), close cell-to-cell contacts could improve virus propagation in suspension cell culture. Therefore, all five cell lines at CCD (2.0 × 10^6^ cells/mL) were supplemented with CaCl_2_ concentrations ranging from 0.3–1 mM to facilitate cell aggregation (Peshwa et al. 1993) and monitored regarding a positive effect on the viral spread. The calcium concentration range was based on a previously conducted CaCl_2_ sensitivity analysis identifying 0–1 mM to be the optimal range for all tested cell lines (data not shown). Furthermore, while virus propagation at higher cell concentrations often results in a reduction of CSVY (“cell density effect”) (Maranga et al. 2003; Kamen and Henry 2004), the use of appropriate medium exchange strategies before infection can overcome this limitation and further increase virus yields (Vázquez-Ramírez et al. 2018). Therefore, the impact of higher cell concentrations (5.0 and 10.0 × 10^6^ cells/mL) at the time of infection on the maximum infectious virus titer was also evaluated. Each condition was assessed by comparing the respective maximum titers with the maximum titer reached in a control infection (Fig. 1) at 2.0 × 10^6^ cells/mL (Table 1). A greater than 1 log increase/decrease in TCID_50_/mL was considered a strong response, greater than 0.3 log increase/decrease a slight response, and smaller than 0.3 log increase/decrease no response, as it was within the dilution error of the TCID_50_ assay. Although calcium supplementation led to increased cell aggregation (data not shown), viral productivity was not increased, as demonstrated by similar viral titers obtained in the presence of 0.3–1.0 mM CaCl_2_ compared to the control. A concentration of 1 mM CaCl_2_ led to a slight decrease in virus yield for HEK293SF-F cells. Although higher cell concentrations led to slight increases (between 0.3 and 1 log) or similar TCID_50_/mL titers for AGE1.CR.pIX, AGE1.CR, BHK-21, MDCK-S8, and MDCK-DM cells compared to the control, increases were all close to the dilution error of the TCID_50_ assay. Increasing the HEK293SF-F cell concentration before infection to 5.0 or 10.0 × 10^6^ cells/mL had a clear negative impact on the maximum TCID_50_/mL.

**Table 1:** Impact of the cell concentration and various CaCl_2_ concentrations at time of infection on the TCID_50_/mL titer

Cell line	5 Mio	10 Mio	0.3 mM CaCl_2_	0.5 mM CaCl_2_	0.9 mM CaCl_2_	1 mM CaCl_2_
AGE1.CR.pIX	** + **	** + **	** ~ **	** ~ **	** ~ **	** ~ **
AGE1.CR	** + **	** ~ **	** ~ **	** ~ **	** ~ **	** ~ **
BHK-21	** + **	** + **	** ~ **	** ~ **	** ~ **	** ~ **
HEK293 Freesytle	**- -**	**- -**	** ~ **	** ~ **	** ~ **	**-**
MDCK Smith-8	** + **	** ~ **	n.d	** ~ **	n.d	** ~ **
MDCK Driving M	** ~ **	** + **	n.d	** ~ **	n.d	** ~ **

Each condition was rated based on the achieved effect on the TCID_50_/mL titer compared to control infection at 2 × 10.^6^ cells/mL: +  + : strong increase above 1 log, + : slight increase above 0.3 log, -: slight decrease below 0.3 log,—-: strong decrease above 1 log, ~ no change (within the 0.3 log dilution error of the TCID_50_ assay). n.d. = not determined

### VSV-NDV release dynamics for different cell lines

In the preceding experiments, we were able to identify the most promising cell lines for further optimization. Based on the maximum TCID_50_/mL, regulatory aspects concerning the cell line as well as production medium (chemically-defined, GMP availability), AGE1.CR.pIX, AGE1.CR, BHK-21, and HEK293SF cells were chosen for further process evaluation. Besides the selection of a suitable host cell, insights into VSV-NDV release dynamics in suspension culture are essential for process optimization. Therefore, MOI studies as well as monitoring of viral spreading and infection ratios via flow cytometry were performed.

**Fig. 3:**
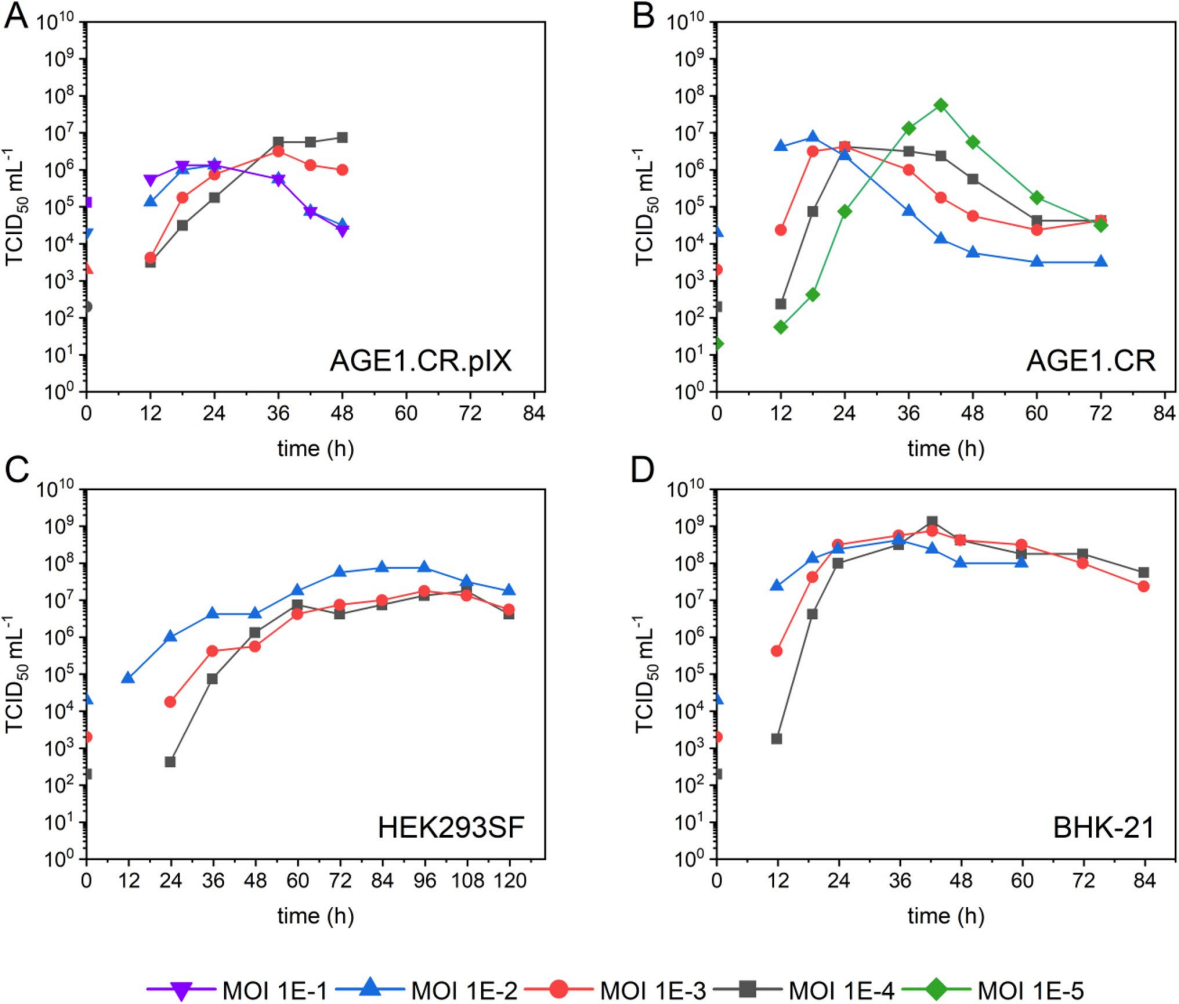
VSV-NDV infection of four suspension cell lines at different MOIs ranging from 1E-5 to 1E-1 in 125-mL shake flasks. Infectious virus titers were calculated by TCID_50_ assay and plotted against the time post-infection. The TCID_50_/mL at 0 hpi was calculated empirically corresponding to the respective MOI. **A** AGE1.CR.pIX in CD-U7 medium, **B** AGE1.CR pIX in CD-U7 medium, **C** HEK293SF in PEM medium, and **D** BHK-21 in PEM medium. This screening experiment was carried out once. For AGE1.CR and AGE1.CR.pIX, additional MOI were tested to verify the possible beneficial impact

To determine the optimum MOI for each cell line and medium, cells were infected at MOIs ranging from 10^−1^ to 10^−5^. As shown in Fig. 3, higher amounts of virus input did not result in higher TCID_50_/mL for AGE1.CR.pIX, AGE1.CR, and BHK-21 cells. However, maximum values were reached earlier (between 18 and 48 hpi). Interestingly, for HEK293SF cells, lower MOIs resulted in a decrease of about one log in TCID_50_/mL compared to other cells infected at an MOI of 10^−2^. Furthermore, peak titers were reached at 96 hpi, independent of the initial virus load. For all other cell lines, the highest overall titers observed were 1.3 × 10^9^ TCID_50_/mL at an MOI of 10^−4^ for BHK-21 cells at 42 hpi, 5.6 × 10^7^ TCID_50_/mL for AGE1.CR cells at an MOI of 10^−5^ at 42 hpi, and 7.5 × 10^6^ TCID_50_/mL for AGE1.CR.pIX cells at an MOI of 10^−4^ at 48 hpi. The infectious units declined after reaching the maximum titer at all conditions, although at different rates. As already observed in Fig. 1, AGE1.CR and AGE1.CR.pIX cells had the steepest reduction of TCID_50_/mL by approximately 1.5–2.0 log within 12 h after reaching the peak, whereas HEK293SF and BHK-21 cells showed slower declines of 0.5–1.0 log within 24 h.

In addition to the maximum infectious virus titers achieved, the fold increase of infectious virions is one of the most important criteria in vaccine production. When comparing the TCID_50_/mL used at TOI with the peak TCID_50_/mL achieved, the fold increase was around 37,000 fold, 2,800,000 fold, 3700 fold, and 6,700,000 fold at the optimal infection MOI for AGE1.CR.pIX, AGE1.CR, HEK293SF, and BHK-21 cells, respectively. Considering the CSVY reached an MOI of 10^−2^, a twofold increase in CSVY for BHK-21, and a fivefold increase for AGE1.CR and a fourfold increase for AGE1.CR.pIX cells were achieved by lowering the MOI to 10^−4^–10^−5^. Only HEK293SF cells displayed a fivefold lower CSVY when decreasing the MOI to 10^−4^.

**Fig. 4:**
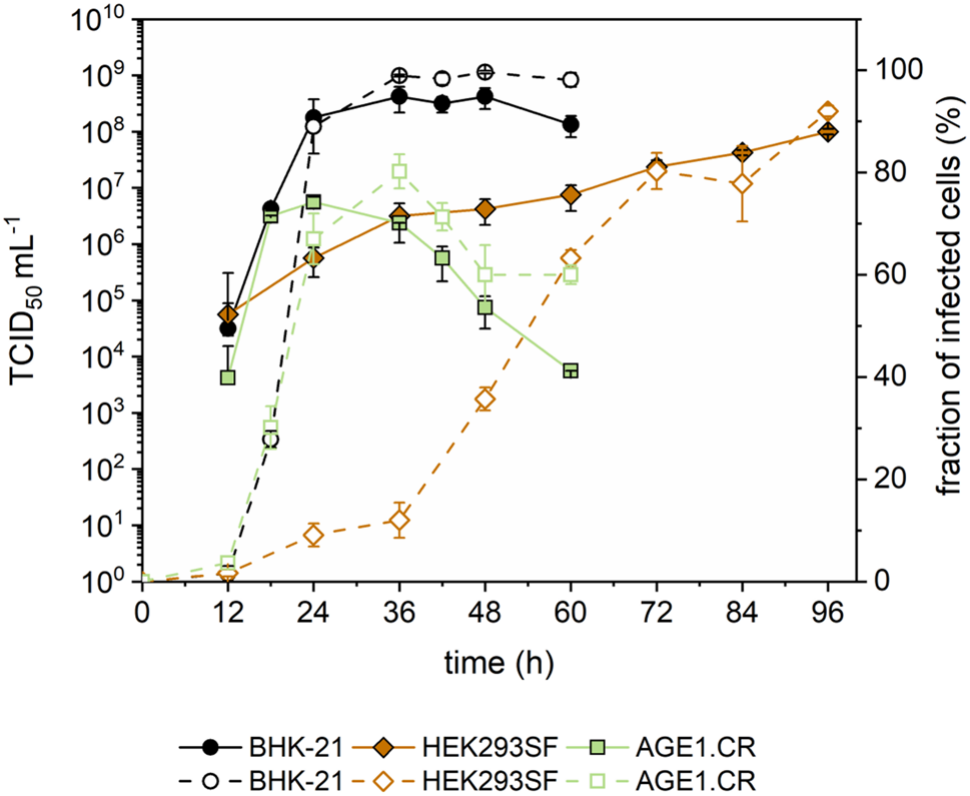
Infection dynamics of VSV-NDV in BHK-21, AGE1.CR, and HEK293SF cells. BHK-21 and AGE1.CR cells were infected at an MOI of 10^−4^, while HEK293SF cells were infected at an MOI of 10^−2^. TCID_50_/mL (full symbols), as determined by TCID_50_ assay, and the fraction of GFP-positive cells determined by FACS analysis (empty symbols) are plotted against the time of infection. Cells were maintained in standard shake flasks and infected at 2 × 10.^6^ cells/mL after a complete medium exchange. The fraction of infected cells was determined with the ImageStream X Mark II. Values are reported as the mean ± standard deviation of two shake flasks

To identify the most suitable candidate cell line, optimal MOIs of the respective cell lines were repeated in three independent shake flasks (Table 2). VSV-NDV-mediated GFP expression in infected cells enabled flow cytometry analysis of the proportion of infected cells from shake flask cultivations of the most promising cell lines BHK-21, HEK293SF, and AGE1.CR (Fig. 4). For all cell lines, the first GFP-positive cells were detected at 12 hpi, although only a small fraction of the cells (1–3%) was GFP positive. For BHK-21 cells, the proportion of GFP-positive cells quickly increased to 89 ± 5% at 24 hpi and reached its maximum of 98.9 ± 0.2% at 36 hpi, indicating a complete infection of all cells. In contrast, the GFP-positive cell fraction only gradually increased for HEK293SF cells with 12 ± 3% infected at 24 hpi and 92 ± 1% at 96 hpi. A strong correlation between the time point of reaching the maximum fraction of infected cells and maximum infectious virus titer was observed for both BHK-21 and HEK293SF cells. Interestingly, a maximum of 80 ± 3% of AGE1.CR cells were infected at 36 hpi, while the VSV-NDV titer peaked earlier (at 24 hpi) but subsequently decreased by 2 log units within one day. Consistent with previous results (Sect. 0), VSV-NDV titers were more or less stable for BHK-21 and HEK293SF cell cultures using a PEM_s_ medium. While a continuous proliferation of HEK293SF cells until 84 hpi could explain the higher virus stability, this was not the case for BHK-21 or AGE1.CR cells.

**Table 2:** VSV-NDV-GFP production in suspension cell cultures. Yields and growth parameters of host cells after infection are shown. Cell concentration, virus decay time, and maximum infectious virus titers were measured in three independent shake flask cultivations

Cell line	Media	Optimum MOI	Max. viable cell concentration (× 10^6^ cells mL^−1^)	TCID_50_ mL^−1^	CSVY* (virions cell^−1^)	Time of max. titer (h)	1 log decay time (h)
BHK-21_sus_	PEM_s_	1 × 10^−4^	5.0 ± 1.0	8.8 × 10^8^ ± 5.6 × 10^8^	140 ± 84	36–42	< 24
HEK293SF	PEM_s_	1 × 10^−2^	7.7 ± 1.0	8.3 × 10^7^ ± 1.4 × 10^7^	11 ± 3	96	< 24
AGE1.CR	CD-U7	1 × 10^−5^	4.3 ± 0.6	4.3 × 10^7^± 2.2 × 10^7^	11 ± 4	42–48	< 12
AGE1.CR.pIX	CD-U7	1 × 10^−4^	3.3 ± 0.2	5.4 × 10^6^ ± 2.2 × 10^6^	2 ± 1	48	< 12

^*^CSVY, cell-specific virus yield

### Adaptation of VSV-NDV to different suspension cell lines

While the seed material, derived from adherent AGE1.CR.pIX cells, propagated well in BHK-21 cells (CSVY reaching 140 TCID_50_/cell), extracellular titers obtained in AGE1.CR.pIX, AGE1.CR, and HEK293SF cells were relatively low (CSVY < 11 TCID_50_/cell for AGE1.CR, < 2 TCID_50_/cell for AGE1.CR.pIX, and < 11 TCID_50_/cell for HEK293SF cells). Therefore, sequential virus passaging over five passages was conducted to evaluate whether adaptation of the virus to the corresponding host cell line could enhance production. It was hypothesized that sequential adaptation would lead to either: (i) an increase in maximum virus titers due to beneficial alterations of the viral fitness or virus stability, and/or (ii) a reduction of the timespan to reach the maximum titers due to a selection of fast-replicating viruses, or alternatively (iii) a decrease of infectious virus titers due to the potential enrichment of defective interfering particles (DIPs) or a selective pressure for virus variants that favor longer persistence in the host cell.

**Fig. 5:**
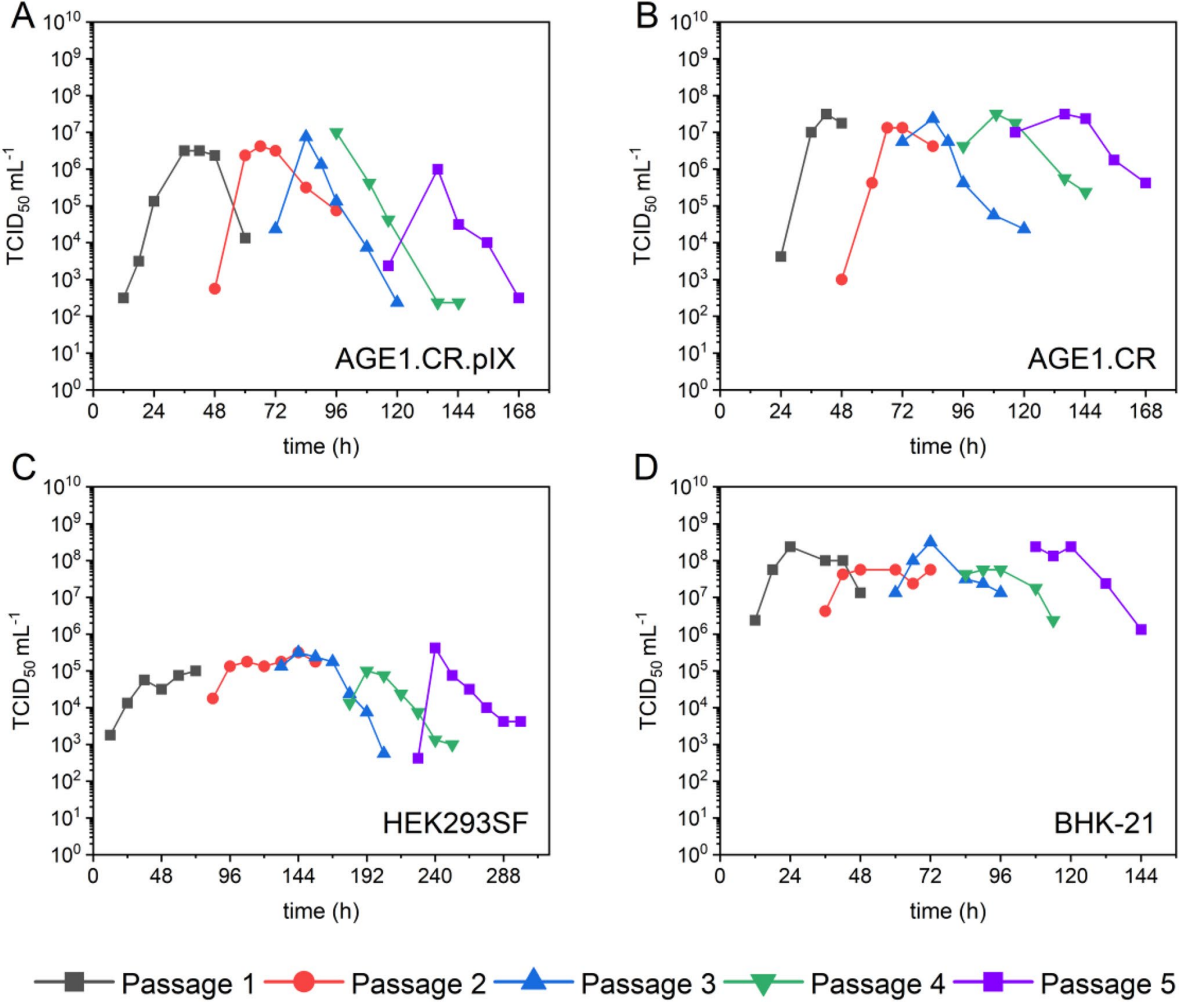
Infectious virus titers during sequential adaptation of VSV-NDV in different suspension cell lines. AGE1.CR.pIX, AGE1.CR, HEK293SF, and BHK-21 cells were cultivated in shake flasks in CD-U7, Dynamis, and PEM medium, respectively. Initial infection occurred during the mid-exponential growth phase at the previously determined optimal MOI. Subsequent virus passaging was performed blindly using a fixed volume. TCID_50_/mL are plotted against the time post-infection. Each color indicates a different virus passage. Shake flask runs were carried out as individual experiments for time, workload, and cost reasons

Despite blind passaging using non-optimized MOI conditions after the first infection, TCID_50_ titers virus remained high in any cell line. For BHK-21 cells, maximum titers of 2.4 × 10^8^ TCID_50_/mL were obtained in the first passage with a CSVY of 80–100 TCID_50_/cell. All subsequent passages achieved maximum titers of around 10^8^ TCID_50_/mL at 24 hpi, although it was observed that later passages showed a faster degradation of infectious virus particles later on (Fig. 5D). Also, AGE1.CR and HEK293SF cells produced similar maximum virus titers over five passages (3.2 × 10^7^ TCID_50_/mL and 3.2 × 10^5^ TCID_50_/mL, respectively) (Fig. 5B,C). In contrast, virus titers in AGE1.CR.pIX cell cultures increased over the first four passages by about 0.5 log, reaching 1 × 10^7^ TCID_50_/mL (Fig. 5A). In addition, the timespan to reach maximum titers was reduced starting from passage three by one day for AGE1.CR and AGE1.CR.pIX cells and by two and a half days for HEK293SF cells. Although maximum titers could not be significantly increased, CSVYs slightly increased for all cell lines over the sequential passaging (data not shown).

### Production of VSV-NDV in BHK-21 and HEK293SF cells in a bioreactor

**Fig. 6:**
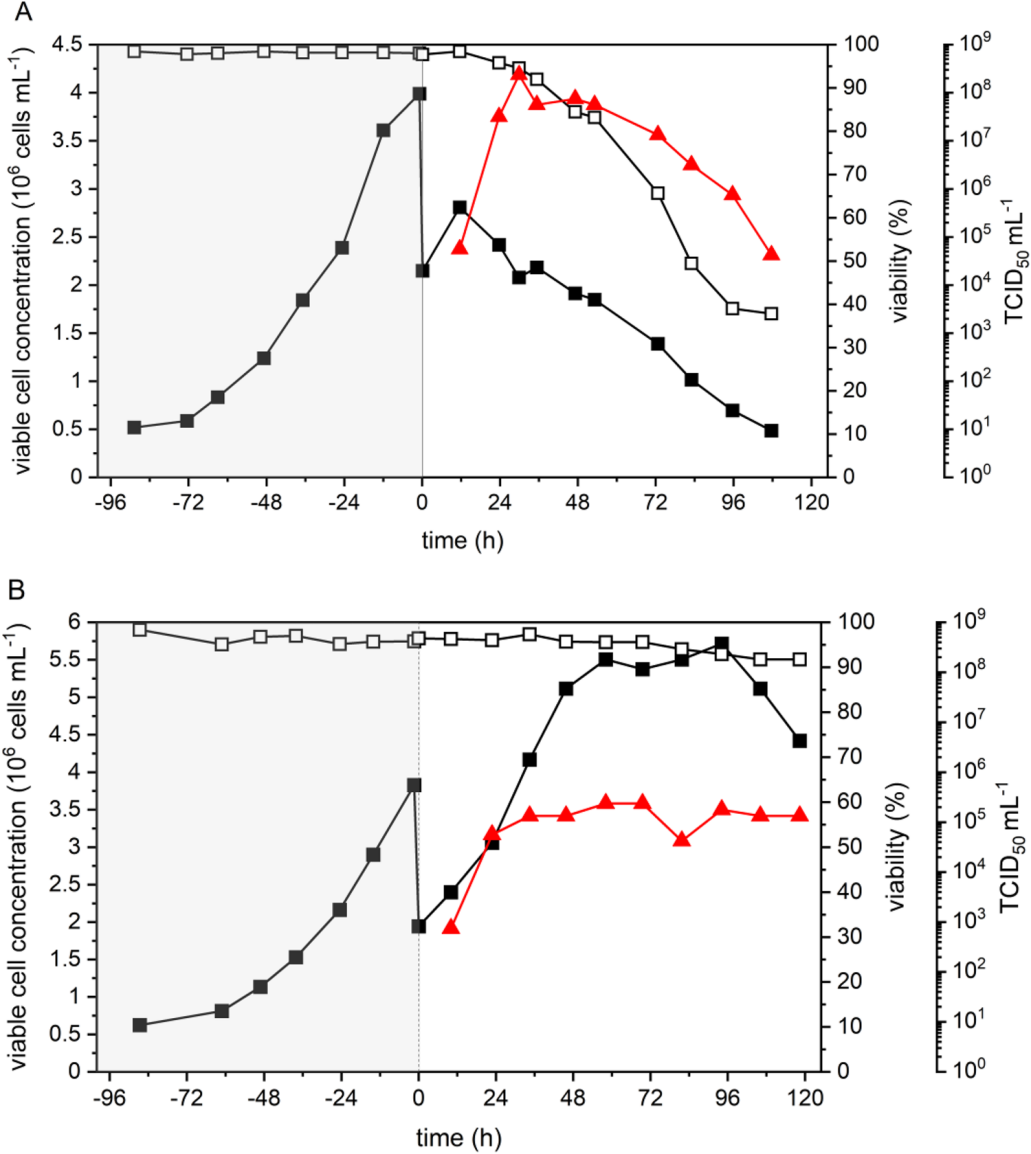
Growth and rVSV-NDV production for BHK-21 and HEK293SF cells in a 1 L bioreactor. BHK-21 (**A**) and HEK293SF cells (**B**) were cultured in a 350 mL supplemented PEM medium. After a cell growth phase of 96 h, cells were infected with VSV-NDV at an MOI of 0.0001 and 0.01, respectively, by adding 350 mL of fresh PEM_s_ medium. Cell concentration (black square), VSV-NDV titer (red triangle), and viability (empty black square) are shown. The dotted vertical line indicates the time of infection; the gray area indicates the cell growth phase. Bioreactor runs were carried out as individual scouting experiments

Due to high yields and stable titers of VSV-NDV in BHK-21 and HEK293SF cell cultures, in the next step, VSV-NDV production was transferred to a 1 L STR (*V*_W_ = 700 mL) to allow for better control of process parameters and to evaluate the potential for further scale-up. Following the cell growth phase of 3.7 days for BHK-21 and 3.6 days for HEK293SF cells in half of the *V*_W_ of PEM_s_ medium, BHK-21 and HEK293SF cells were infected by addition of 350 mL fresh medium at an MOI of 10^−4^ and 10^−2^, respectively. Before infection, cell-specific growth rates (*µ*_mean_) of 0.027 1/h for both BHK-21 and HEK293SFcells were observed. After infection, BHK-21 cell concentration continued to increase for 12 hpi with a *µ*_mean_ of 0.023 1/h, followed by a decrease 12 h later due to the cytopathic effect caused by viral replication. Cell viability remained higher than 90% for 36 hpi and rapidly declined afterward (Fig. 6A). The pH remained stable and was efficiently controlled at 7.2 ± 0.1 (data not shown). In contrast, HEK293SF cell concentration continued to increase until 96 hpi with a *µ*_mean_ of 0.018 1/h, reaching a maximum cell concentration of 5.7 × 10^6^ cells/mL. Cell viability was higher than 90% for the entire cultivation (Fig. 6B). The pH was efficiently controlled at 7.2 ± 0.2, slightly increasing at the end of the cultivation (data not shown).

For BHK-21 cells, virus production peaked at 30 hpi with 2.4 × 10^8^ TCID_50_/mL (CSVY of 84 TCID_50_/cell). As before, the TCID_50_/mL slowly declined thereafter by 0.5 log within 24 h. Overall, maximum virus titer, CSVY, and virus stability were similar to the reference infections in shake flasks (Table 2), where no complete medium exchange was performed before infection. Although HEK293SF cells reached high virus titers in shake flasks (Table 2), this was not observed for the 1 L STR culture. At 58 hpi, a maximum infectious titer of only 2.4 × 10^5^ TCID_50_/mL was reached, which corresponds to a 2.5 log reduction compared to previous shake flask runs. Due to the low virus titer and prolonged cell proliferation after infection, only a low CSVY of 0.04 TCID_50_/cell was obtained.

**Fig. 7:**
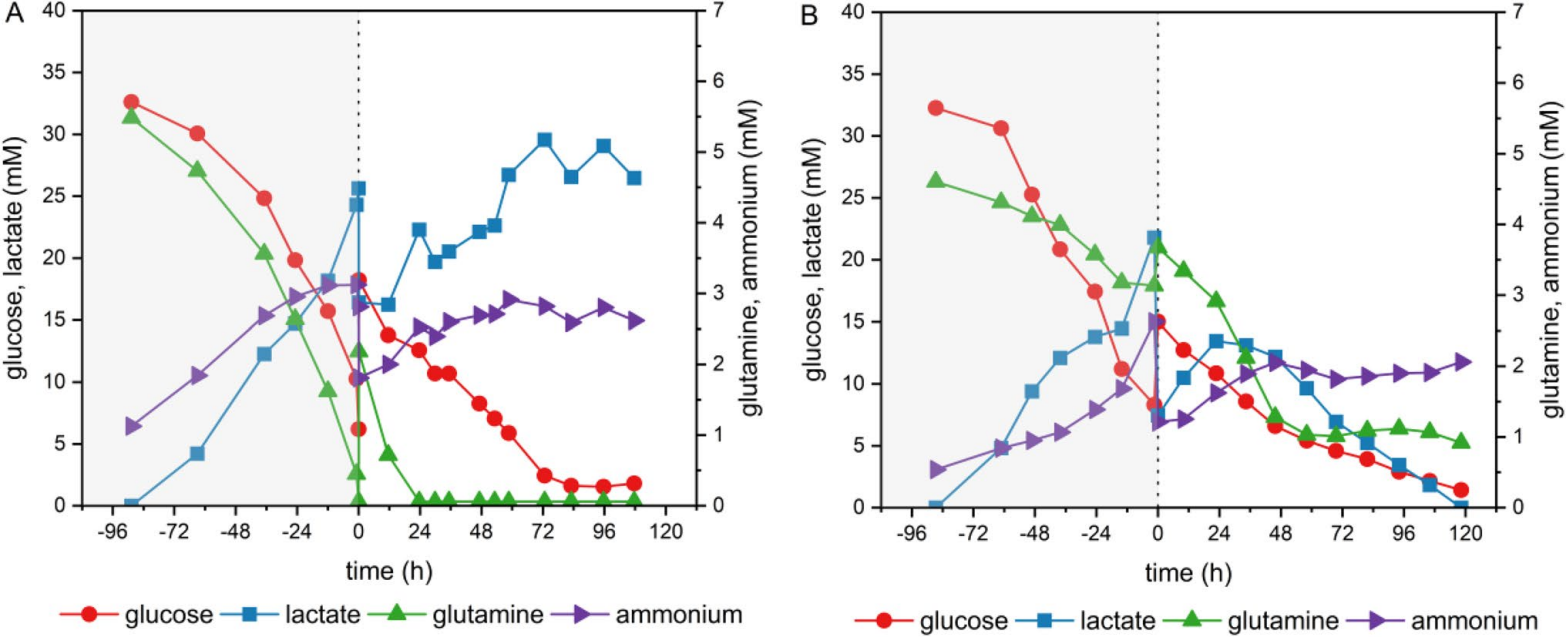
Time course of metabolites for BHK-21 and HEK293SF cells in a 1 L bioreactor. BHK-21 (**A**) and HEK293SF cells (**B**) were cultured in 350 mL supplemented PEM medium. After a cell growth phase of 96 h, cells were infected with VSV-NDV at an MOI of 0.0001 and 0.01, respectively, by adding a 350 mL of fresh PEM_s_ medium. Glucose, lactate, ammonium, and glutamine concentrations were measured with the BioProfile® 100 Plus (Nova Biomedical, USA). The dotted vertical line indicates the time of infection; the gray area indicates the cell growth phase. Bioreactor runs were carried out as a single scouting experiment

As an indication of the cell’s metabolic activity, the concentration of the main carbon and energy sources, glucose and glutamine, as well as their metabolic by-products, lactate, and ammonium, were monitored before and after infection with VSV-NDV (Fig. 7). As expected, no limitation of glucose was observed for either cell line during the entire cultivation run. Despite high glutamine concentrations in the PEM_s_ medium, depletion occurred at 24 hpi for BHK-21 cells. Moreover, the addition of a new medium after infection could not prevent the accumulation of ammonium (2–3 mM) and lactate (20 mM) to high (and typical toxic) concentrations. Interestingly, for HEK293 cells, glutamine was sufficiently maintained during the entire cultivation, and ammonium, as well as lactate, remained below critical values during the virus production phase.

**Fig. 8:**
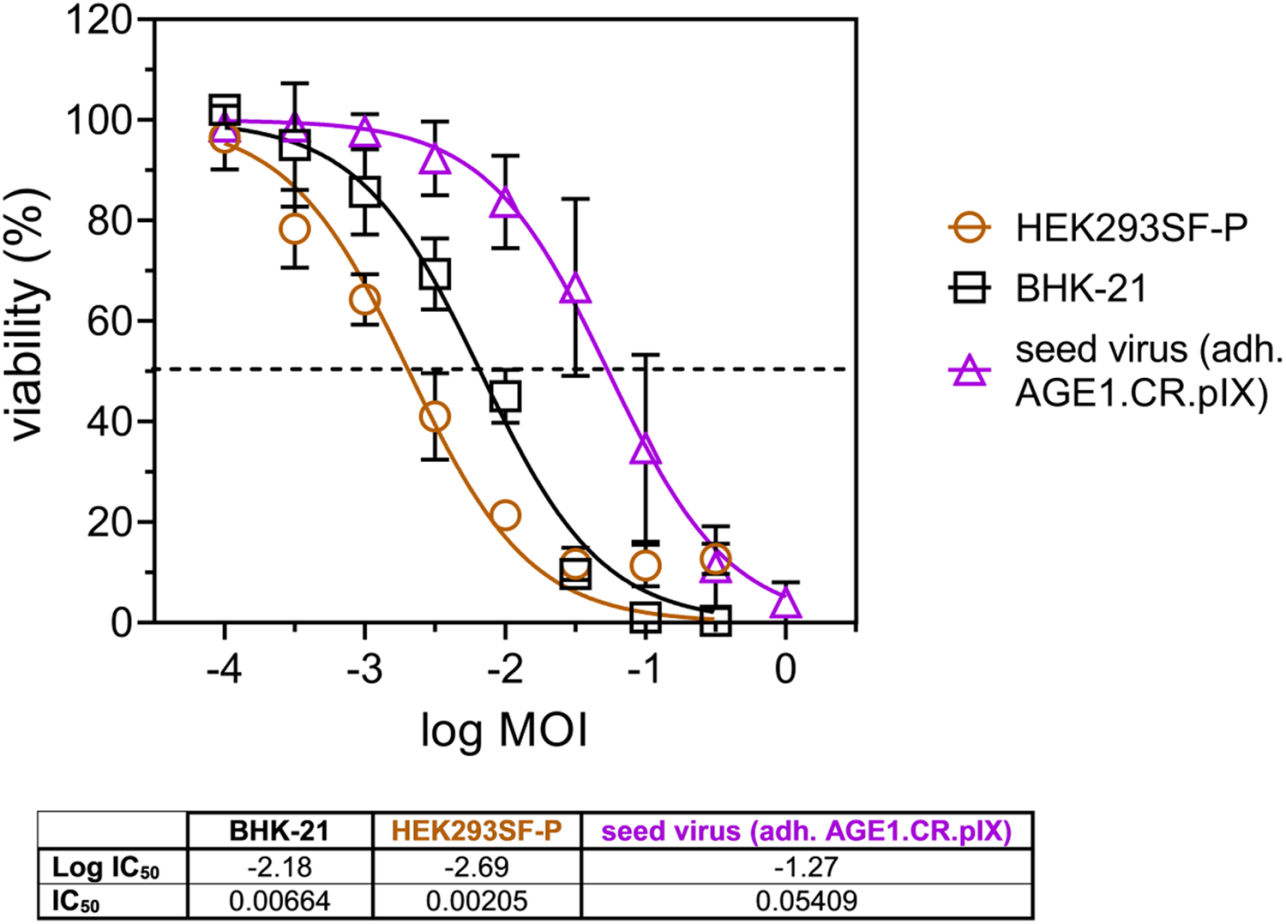
Oncolytic potency of VSV-NDV produced in a 1 L STR. The cancer cell killing potential of VSV-NDV harvested from the individual bioreactor cultivations was assessed by IC50 assay in human HCC cells (Huh7) and compared to the initial seed stock used for infection. Cell viability was monitored by CellTiter-Glo Assay (Promega). The graph shows dose–response curves after non-linear regression analysis to calculate the individual IC_50_ and logIC_50_ values shown in the table below. Values are reported as the mean ± standard deviation of technical replicates, with *n* = 3 for seed stock and bioreactor samples, respectively

Next, we analyzed whether the process conditions in a STR affected the ability of the virus to induce oncolytic killing in susceptible Huh7 cells using the IC_50_ potency assay. Samples from the bioreactor were compared to a seed virus stock generated in adherent AGE1.CR.pIX cells. As indicated in Fig. 8, VSV-NDV harvested from the 1 L STR of HEK293SF or BHK-21 cell cultures had a similar oncolytic potential in Huh7 cells compared to the control. Remarkably, the dose required to induce 50% cancer cell cytotoxicity was more than 1 log lower for VSV-NDV produced in suspension bioreactor cultures.

## Discussion

Currently, 80 clinical trials with oncolytic viruses are registered at ClinicalTrials.gov, and one such agent, an oncolytic herpes virus against melanoma (IMLYGIC®, Amgen Inc.) has obtained market approval in the US and Europe in 2015. As such novel therapies gain traction, the need for robust, efficient, and scalable manufacturing processes for oncolytic viruses becomes evident. The aim of this study was to explore the applicability of different suspension cell lines for the production of rVSV-NDV. As a fusogenic oncolytic virus, it poses unique challenges for manufacture because it kills host cells rapidly via syncytia formation and replicates to relatively poor yields in cell cultures. Previous results with anchorage-dependent AGE1.CR.pIX cells (Abdullahi et al. 2018) are not easily translated to large-scale manufacturing and process intensification (Genzel and Reichl 2009; Gallo-Ramírez et al. 2015). Therefore, cell lines already adapted to growth in suspension (Supplement Table S1) and have been used to produce other viruses in bioreactors (Jordan et al. 2009; Lohr et al. 2009, 2010; Chu et al. 2009; Grieger et al. 2016; Nikolay et al. 2018), were tested in this study against oncolytic VSV-NDV.

### Screening for host cell selection

All tested cell lines (of anatine, rodent, canine, and human origin) were permissive to VSV-NDV but differed in cell line-specific virus growth characteristics, with maximum virus yields spread over 3 orders of magnitude (Fig. 1). The envelope of VSV-NDV contains two associated glycoproteins from NDV that mediate viral entry and cell-to-cell fusion: the hemagglutinin-neuraminidase (HN) and a modified fusion (F) protein for an enhanced cell–cell fusion potential (Sánchez-Felipe et al. 2014; Abdullahi et al. 2018). HN recognizes and binds to sialoglycoconjugates (Sia) on the cell surface, specifically α2,6-linked Sia, which appears to be present at comparably low levels in all tested cell lines (Genzel and Reichl 2009; Lohr et al. 2012; Chu et al. 2019). The observed differences in virus replication indicate that receptor abundance alone may not be a decisive factor in determining yields also of this replication-competent virus in a given host cell. However, it should be noted that VSV-NDV may utilize additional or alternative cell surface receptors for infection, as the modes of binding, entry, and fusion of this novel hybrid vector are not yet fully understood.

VSV-NDV has an inherent cancer cell specificity but was also shown to replicate in adherent, immortalized cell lines, e.g., AGE1.CR.pIX and BHK-21 cells, with modest titers of 10^6^ TCID_50_/mL (Abdullahi et al. 2018). In comparison, infections in suspension AGE1.CR, MDCK, HEK293SF, and BHK-21 cells (Fig. 1) yielded 10- to 100-fold increases in TCID_50_/mL.

The heterogenous curves in Fig. 1 illustrate some of the counteracting processes during productive viral infections. Virus replication usually is strong in host cells in the log phase of proliferation when the supply of nutrients is not limiting. Here for VSV-NDV, maximum titers were achieved in all cell lines ± 12 h after peak cell concentration. As the productivity of infected cells declines, the stability of virions becomes increasingly important. Productivity inversely correlates with viability decreased at different rates for the various cell lines. A pronounced decline was observed in AGE1.CR, AGE1.CR.pIX, and MDCK-DM cultures start about 18 hpi. CPE was strong also in BHK-21, and to a lesser degree in HEK293SF-D cultures.

The stability of virions can be affected by the absorption of cellular debris and the release of cellular proteases after virus-induced cell lysis. Depending on the culture environment (especially the capacity of the medium to buffer pH changes and content of hydrolysates, protein, and divalent cations), such effects can be more or less detrimental to the infectious units. In contrast to the avian cells, although the viability of BHK-21 and MDCK-DM cells clearly declined by 36 hpi, the TCID_50_/mL remained relatively stable.

Previous studies showed that VSV-NDV primarily spreads through cell-to-cell contacts supported by the formation of syncytia in adherent cell cultures (Abdullahi et al. 2018). This suggests that aggregate formation in suspension cell cultures could also promote viral spread and productivity. Indeed, for MDCK cells growing as aggregates (MDCK-S8), we observed a 1 log higher TCID_50_/mL compared to single-cell suspensions (MDCK-DM). Nevertheless, no syncytia formation was observed in suspension cultures for any cell line regardless of the used medium, and cell aggregation, in general, did not seem to be the most important factor for high VSV-NDV yields.

While efficient oncolytic virus manufacture in suspension cultures was our primary aim, the quality of the VSV-NDV product was an obvious concern. We confirmed that VSV-NDV stocks produced in the suspension cell lines investigated are not compromised in their oncolytic potency compared to virus stocks produced in adherent cultures. Only virus harvested from MDCK cells in Smif8 media caused poor oncolysis in susceptible Huh7 cells, although the titers were superior compared to the Driving medium. This observation underlines the importance of cultivation media and might point to a change in viral integrity or virus aggregation negatively influencing oncolysis. In contrast, the improved potency values for rVSV-NDV produced in AGE1.CR.pIX and HEK293SF-P cells might be explained by superior viral integrity or possibly by beneficial changes in the ratio of infectious virions to DIPs. As further discussed below, the impact of DIPs on tumor cell lysis and the CPE is being investigated in ongoing studies.

### Effect of cell concentration and CaCl_2_ supplementation on VSV-NDV production

Our data suggest that cell-to-cell contact is not critical for the production of VSV-NDV in suspension cultures. This was further confirmed as induction of cell clumping via supplementation of CaCl_2_ did not lead to a measurable increase in infectious virus titers (Table 1), taking into account the dilution error of the TCID_50_ assay. Although cell-to-cell proximity should allow for a more efficient viral spread, virus production was not increased. This is in line with observations made for cancer cells, which demonstrated that tumor cell killing with VSV-NDV was similar to that of the parental VSV vector, despite virus replication of the hybrid virus being substantially attenuated compared to the parental VSV (Abdullahi et al. 2018). Although this is one of the safety benefits of VSV-NDV and other fusogenic OVs (Krabbe and Altomonte 2018), it also causes manufacturing challenges. In fact, as mentioned before, this was one of the rationales for the establishment of a suspension culture-based VSV-NDV production process. The exact mechanisms of and dependencies on syncytial cell death and cell-to-cell spread are not yet fully understood for VSV-NDV, but they could be a potent parameter to further optimize viral replication during production. Moreover, closer cell-to-cell contacts induced by higher cell densities at the time of infection also did not result in a significant increase in titers for AGE1.CR, AGE1.CR.PIX, MDCK, and BHK-21 cells. Indeed, a substantial decrease in yields was observed for HEK293SF cells at higher cell densities. The latter is most likely due to nutrient limitations and/or inhibitor accumulation, the so-called “cell density effect,” commonly observed for adenovirus production in HEK293 cells (Kamen and Henry 2004).

### VSV-NDV release dynamics for different cell lines

We next tried to improve viral titers by determining the optimal MOI. All cell lines, except HEK293SF, demonstrated a clear benefit of using lower MOIs (10^−4^–10^−5^) (Fig. 3). Interestingly, for a lentogenic NDV dependent on an exogenous protease for replication in AGE1.CR.pIX cultures, high yields were obtained with an MOI as low as 10^−7^ (Jordan et al. 2016). Accordingly, options for further reductions in MOI should be tested in subsequent optimization studies to investigate whether the maximum infectious titers can be increased even more.

However, in addition to viral yield and titer, the ratio of the viral genome and subgenomic copies to the actual infectious units is an important parameter (Fernandes et al. 2016). A recent study with wild-type VSV reported increased accumulation of noninfectious viral genomes at lower MOIs of 10^−3^–10^−4^, mainly caused by loss of infectious activity due to longer production times (Kiesslich et al. 2020). In follow-up studies, this aspect should be critically assessed to evaluate the potential effect of a reduction of MOI on the final product quality.

Variations in the abundance of DIPs as a consequence of the manufacturing process should also be monitored and carefully discussed in terms of downstream therapeutic implications. DIPs, by definition, interfere with the replication of infectious viral progeny. However, because DIPs may stimulate type I IFN-mediated innate immune responses (Yang et al. 2019; Rand et al. 2021), they can contribute to an important therapeutic mechanism. Recent studies have highlighted antitumor effects of defective viruses, for example, via induction of cell-selective apoptosis in cancer cells through RIG-I-dependent signaling as well as activation of antitumor immunity induced by dendritic cells and T cells (Yang et al. 2019). As the accumulation of DIPs is more likely to occur upon infection at high MOIs, infection at low MOIs could lead to a reduction in DIP concentration (Heldt et al. 2013; Hein et al. 2021), thereby reducing the possible indirect antitumor effect, but potentially enhancing the direct oncolytic effect. As the competing mechanisms regarding the effects of contaminating DIPs in OV drug products are quite complex, this is beyond the scope of the current investigation. Further studies quantifying infectious virion to DIP ratios as well as characterizing their relevance during oncolysis and their therapeutic efficacy should be performed for optimization of virus-based cancer therapeutics.

In the next step, virus infection dynamics were studied in AGE1.CR, HEK293SF, and BHK-21 cell populations using flow cytometry (Fig. 4). The low percentage of infected cells during the first 12 hpi was mainly due to the low virus input (MOI 10^−2^ or 10^−4^). Despite a 100-fold higher initial virus input for HEK293SF cells, the fraction of infected cells increased only slowly over the entire cultivation period, reaching a maximum of 96 hpi. This suggests a delay in the onset of intracellular replication. Especially with respect to increased shear forces due to stirring of suspension cultures, it was also speculated that VSV-NDV may spread primarily through single-cell infection rather than fusion, resulting in a slower gradual infection of the cell population. This could be the reason why lower MOIs led to lower TCID_50_/mL in HEK293SF, as only a fraction of the cells could be infected.

The cell-specific differences in optimal MOI may also be explained by varying levels of efficiency in interferon (IFN) signaling in the respective cell lines. As an RNA virus, VSV-NDV is extremely sensitive to the antiviral actions of type I IFNs (Abdullahi et al. 2018). Upon infection with very low MOIs, IFN-functional cells would quickly sense the infection, trigger IFN signaling cascades, and alert neighboring, still uninfected cells to shift into an antiviral state. Thereby, virus replication in cells that become infected at later stages in the manufacturing process would be attenuated. Consistent with such a mechanism, complete infection of the AGE1.CR cells population was not achieved, and a maximum titer was encountered when only 70% of the population was infected. This indicates an efficient de novo generation of infectious virus, but induction of cell death before the whole population could be infected. Such a course is typically observed in highly susceptible adherent cancer cells, such as Huh7 and HepG2 cells (Abdullahi et al. 2018). From a virus production perspective, it appears favorable to achieve a continuously increasing population of OV-infected cells rather than losing the cell-substrate due to fast oncolysis before high titers are reached. Accordingly, the influence of the MOI on the percentage of infected cells and virus yields should be further investigated. Alternatively, yields may also be higher in processes where interferons may be removed by perfusion cultivation.

### Adaptation of VSV-NDV to different suspension cell lines

Adaptation of a virus to a host cell by serial passaging is likely to improve its replication dynamics. For example, an earlier onset of influenza virus release during virus adaptation has been described in Vero and HEK293 cells (Le Ru et al. 2010; Genzel et al. 2010) and for lentogenic NDV in CR.pIX cells (Jordan et al. 2016). In our study with VSV-NDV, serial passaging for five cycles also yielded an adapted oncolytic virus population with an earlier onset of virus accumulation in the supernatant. With the adapted virus seed, maximum titers were obtained one day faster for AGE1.CR and AGE1.CR.pIX cells and 2.5 days earlier for HEK293SF cells (Fig. 5). While TCID_50_/mL was not increased significantly for any cell line, virus stability was drastically reduced. This is likely due to the enhanced CPE of the adapted virus, which reduced cell proliferation and led to increased cell lysis after infection compared to the non-adapted virus (Supplement Figure S2). We speculate that the shortened time span to reach maximum titers is a result of more efficient intracellular virus replication and shows that adaptations can be a crucial parameter to improving OV process performance.

A previous study described improved replication for a recombinant VSV virus expressing a chimeric Sindbis glycoprotein after low-MOI infection and adaptation over 15 passages (Gao et al. 2006). This was mainly credited to an increased expression of the glycoprotein on the viral surface, which resulted in higher infectivity and greater stability (Gao et al. 2006). Theoretically, an elevated expression of the HN and F-proteins on the rVSV-NDV surface could also lead to higher infectivity, but quantitative data on VSV-NDV glycoprotein expression levels are not yet available. Furthermore, as titers did not increase substantially and virus stability appeared to be reduced after repetitive passaging (Fig. 5), other mechanisms may apply under the conditions investigated here.

Furthermore, for VSV, it is known that lower passage numbers (1–8) can maintain viral fitness but are suboptimal for sustaining gains of function, and the passaging MOI is likewise critical for efficient adaptation (Gao et al. 2006; Thompson and Yin 2010). Moreover, as the passaging time span was reduced, lower (only retrospectively defined) MOIs were used in subsequent passages due to lower virus titers at the respective time point. The observed unfavorable adaptation curves in Fig. 5 can be interpreted by generation and fast accumulation of DIPs that negatively affect infectious titers and counteract adaptation. They may also be caused if passaging with a low virus load leads to a phenomenon called Muller’s ratchet, the progressive reduction of viral fitness when detrimental mutations become fixed in a viral population, thereby decreasing overall fitness (Chao 1990; Gao et al. 2006; Sanjuán and Domingo-Calap 2019). Generation of DIPs is favored by higher MOIs, whereas effects by Muller’s ratchet are facilitated by low MOIs. Retrospective calculation of MOI over the five passages revealed broad cell line-dependent ranges: (i) BHK-21 cells 10^−2^–10^−3^, (ii) AGE1.CR cells 10^−4^–10^−7^, (iii) AGE1.CR.pIX cells 10^−4^–10^–5^, and (iv) HEK293SF cells 10^−2^–10^−6^. This variation in MOI could be resolved by switching from a fixed-volume-based passaging to a fixed-MOI passaging. In support of such an approach, Thompson et al. reported that changes in virus yield over the first six passages in fixed-MOI (0.1) passaging of wild-type VSV on adherent BHK-21 cells were prevented (Thompson and Yin 2010). Nevertheless, from the adaptation experiment performed with VSV-NDV, we are optimistic that, with a careful choice of MOI and timespan as soon as a final production process is defined, we can maximize viral fitness and adapt the oncolytic virus to the most favorable producer cell line. Next-generation sequencing of virus populations obtained by passaging could also be instructive in identifying virus adaptation at a genomic level and may allow targeted improvements.

### Production of VSV-NDV in BHK-21 and HEK293SF cells in a bioreactor

Based on promising oncolytic virus titers obtained in small-scale shake flasks and the potential for process intensification, we used BHK-21 and HEK293SF cells as a model to scale the process to 1 L STR. The use of benchtop bioreactors resulted in slightly lower TCID_50_/mL titers (between 0.3 and 1 log) for BHK-21 cells and drastically reduced titers for HEK293SF cells compared to shake flask cultivations with similar virus dynamics. The addition of a fresh supplemented PEM medium at TOI could not prevent glutamine depletion and substantial accumulation of lactate and ammonium for BHK-21 cells. High concentrations of lactate (above 20 mM) and ammonium levels of 2–3 mM have been shown to negatively impact virus vaccine production (Schneider et al. 1996). However, these limits were not exceeded for the HEK293SF cell culture. Interestingly, a decrease in lactate concentration was observed starting at 24 hpi once the glucose level dropped below 10 mM, demonstrating the ability of HEK293SF cells to use lactate as a carbon source after glucose depletion (Liste-Calleja et al. 2015). We suspect that the medium addition at TOI did not allow to provide sufficient nutrients and dilute spent metabolites for HEK293SF cells. This so-called cell density effect is well known, even at low cell densities (above 1 × 10^6^ cells/mL) for adenovirus production in HEK293 cells (Kamen and Henry 2004). Despite an unfavorable metabolic state of BHK-21 cells in the STR, a relatively high CSVY of 84 was reached. In light of future preclinical and clinical studies using VSV-NDV and assuming a dose requirement of at least 1 × 10^8^ TCID_50_/patient, this CSVY would allow the production of approximately 1800 dose equivalents per 1 L run (disregarding losses during downstream processing at this time of the development).

The small number of replicates for most of the experiments and the large reported error of the TCID_50_ assay are limitations of this study. Because the scope of this study was to determine whether single-cell suspension cultures can be used at all to produce fusogenic oncolytic viruses at high titers in a potentially scalable production process, the focus was on the screening process. For this reason, no statistical analysis was carried out, and with exception of the evaluation of the optimum MOI, reproducibility was not further investigated. Results obtained here identify cell line-dependent challenges for upstream processing and should help to guide scale-up developments. To overcome metabolic limitations and augment the production of the fusogenic oncolytic virus, higher cell concentrations in a fed-batch or perfusion mode cultivation should be considered. Perfusion systems utilizing a variety of cell retention devices to achieve higher cell concentrations and higher virus titers have been used successfully for virus production (Gränicher et al. 2020; Coronel et al. 2020). For the production of cell contact-dependent fusogenic oncolytic viruses, high cell density suspension cultures have not been described so far. However, we believe that such approaches hold much promise for further productivity improvements.

Here, we defined cell substrate, media, and MOI as critical process parameters for VSV-NDV production. Beyond achieving high infectious virus titers, a comprehensive screening and testing of the produced material are required before a final decision regarding process conditions, including cell line and media selection, can be made for clinical-grade manufacturing. This concerns, for example, studies in mouse and rat tumor models and serum stability investigations. Moreover, further investigations involving multiple replicates to determine reproducibility for selected conditions are critical.

In summary, this work represents an important step forward in the establishment and optimization of efficient and scalable manufacturing processes for virotherapeutic drug development, particularly as OVs make their way into broader clinical applications. In addition to optimization of infectious yields, the influence of process parameters on the oncolytic activity of virus preparations (possibly containing contaminating DIPs) needs to be confirmed by studies in preclinical tumor models.

## Supplementary Information

Below is the link to the electronic supplementary material.

Supplementary file1 (PDF 385 KB)

## Data Availability

Data is available in the article’s Supplementary material. Additional data is available on request from the authors. The data that support the findings of this study are available from the corresponding author, Yvonne Genzel, upon reasonable request.
