# Supplementary material for: Cell-line screening and process development for a fusogenic oncolytic virus in small-scale suspension cultures
Source: Appl Microbiol Biotechnol. 2022 Jun 29;106(13-16):4945–61. doi: 10.1007/s00253-022-12027-5 (PMC9329169; doi:10.1007/s00253-022-12027-5)
Supplement: Supplementary file 1 — Supplementary file1 (PDF 385 KB) [file 253_2022_12027_MOESM1_ESM.pdf]

Journal: “Applied Microbiology and Biotechnology”

Manuscript Title: “Cell-line screening and process development for a fusogenic oncolytic virus in small-scale suspension cultures”

Sven Göbel[1], Fabian Kortum[2], Karim Jaén Chavez[2], Ingo Jordan[3], Volker Sandig[3], Udo Reichl[1,4], Jennifer Altomonte[2], Yvonne Genzel[1*]

[1]Bioprocess Engineering, Max Planck Institute for Dynamics of Complex Technical Systems, Sandtorstr. 1, 39106 Magdeburg, Germany

[2]Department of Internal Medicine II, Klinikum rechts der Isar, Technische Universität München, Germany

[3]ProBioGen AG, Herbert-Bayer-Str. 8, 13086 Berlin, Germany

[4]Chair for Bioprocess Engineering, Otto-von-Guericke-University Magdeburg, Universitätsplatz 2, 39106 Magdeburg, Germany

*Corresponding author
Yvonne Genzel
Email address: genzel@mpi-magdeburg.mpg.de

Supplementary Material

Table S1: Overview of suspension cell lines tested for the production of VSV-NDV.

Abbreviation	Full name	Species	Tissue	Supplier
BHK-21	Suspension cell growth adapted BHK-21	Syrian golden hamster	Kidney	In house (Nikolay 2014)
AGE1.CR	-	Muscovy duck	Primary retina cell	PBG (Jordan et al. 2009)
AGE1.CR.pIX	-	Muscovy duck	Primary retina cell	PBG (Jordan et al. 2009)
HEK293SF	Human embryonic kidney SF-3F6	Homo sapiens	Embryonic kidney cell	UAB/NRC
MDCK	Suspension cell growth adapted Madin Darby canine kidney CL-34	Dog	Kidney epithelial cell	In house (Lohr et al. 2010)

PBG = ProBioGen AG Berlin, Germany, UAB= Autonomous University of Barcelona, NRC= National Research Council of Canada

Table S2: Cell line-specific passaging times and volumes used for the sequential adaptation of VSV-NDV.

Cell line	Passage 1	Passage 2-5	Transferred volumes (µL)
BHK-21	24 hpi	24 hpi	10
AGE1.CR	24 hpi	24 hpi	10
AGE1.CR.pIX	36 hpi	24 hpi	10
HEK293SF	72 hpi	48 hpi	250

hpi = hours post infection

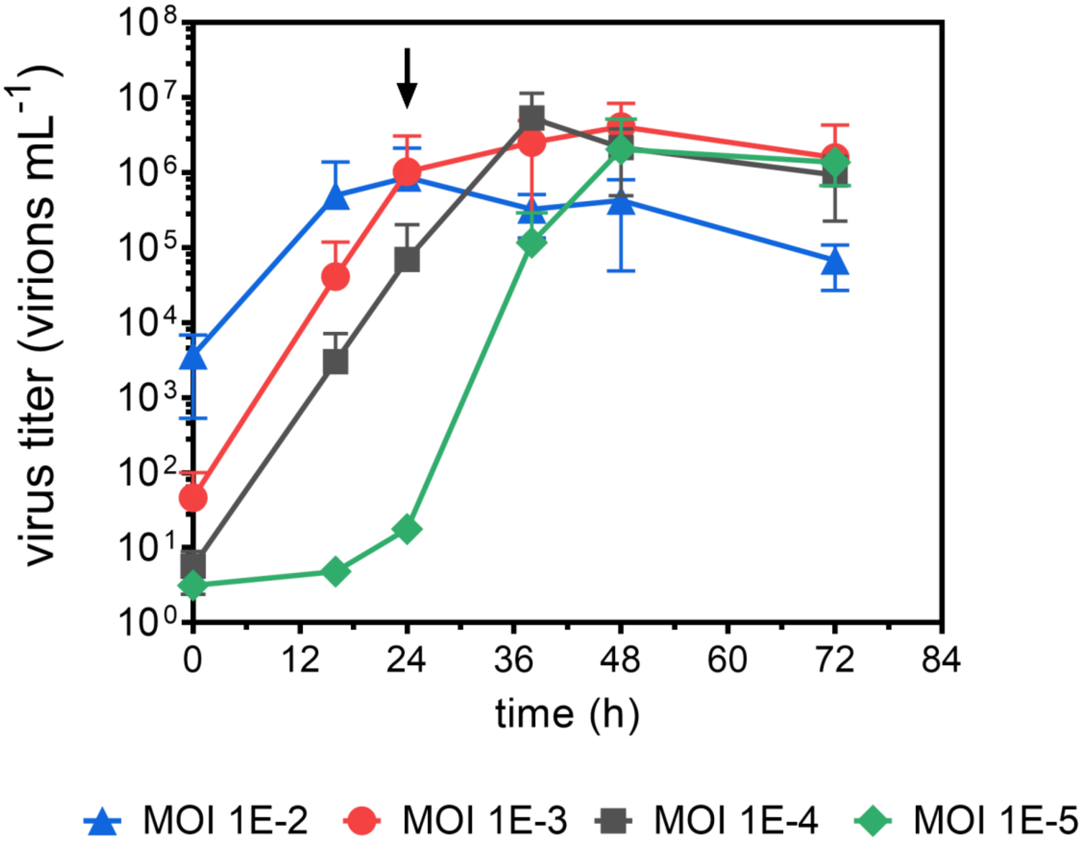

Figure S1 Growth kinetics in adherent AGE1.CR.pIX cells. Adherent AGE1.CR.pIX cells were seeded onto 150 cm^2 tissue culture dishes (#93150, TPP, Switzerland) and infected at a cell concentration of $1x10^5$ $cells/cm^2$ at the respective MOI with VSV-NDV using 22 mL final volume (serum-free DMEM-F12). Virus growth dynamics was monitored by measuring the infectious virus titer in the culture supernatant by the $TCID_{50}$ assay. The graph show mean titers +/- SD from three biological replicates. The arrow marks the peak titer of MOI 0.01 infections, which were the samples used in subsequent potency measurements (Figure 2).

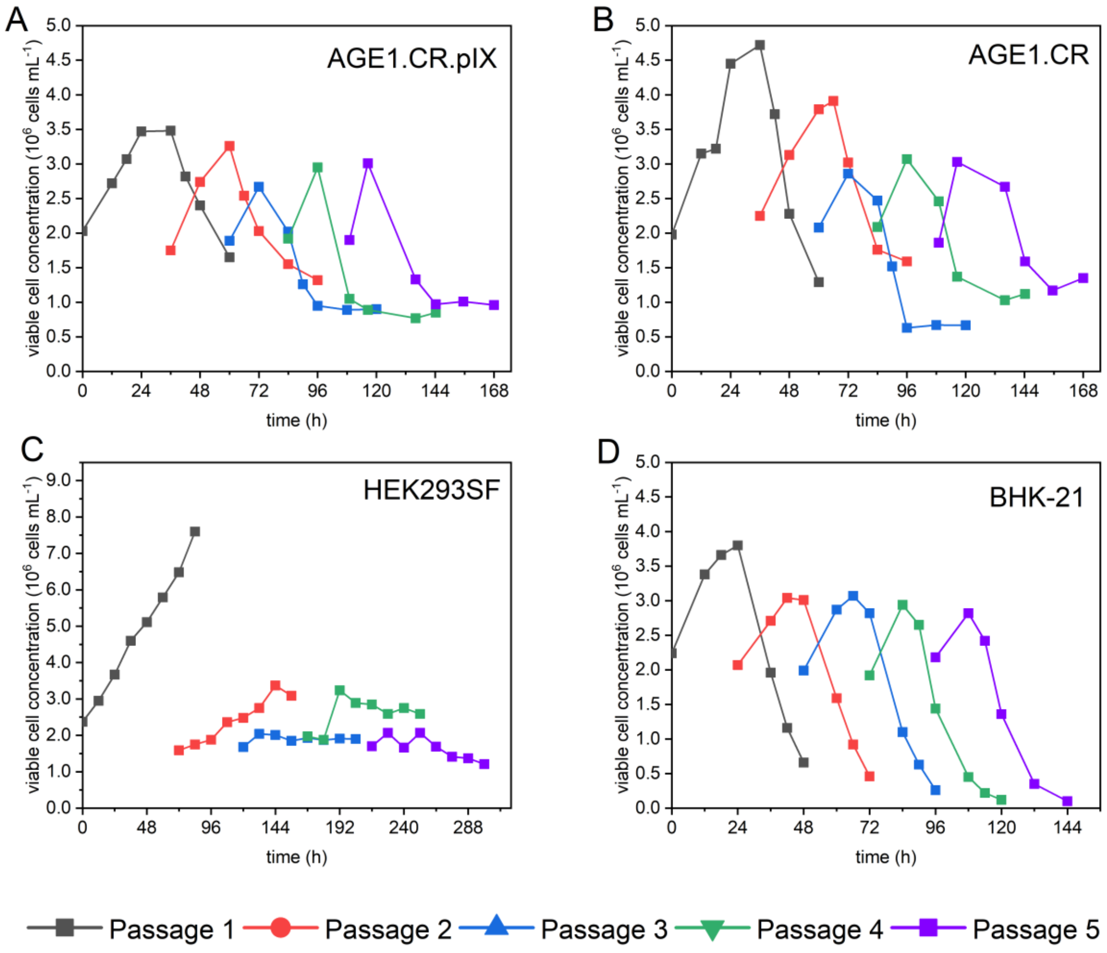

Figure S2 Sequential adaptation of VSV-NDV in different suspension cell lines. AGE1.CR.pIX, AGE1.CR, HEK293SF and BHK-21 cells were cultivated in shake flasks in CD-U7, Dynamics, and PEM_S medium, respectively. Initial infection occurred during the mid-exponential growth phase at the previously determined optimal MOI. Subsequent virus passaging was performed blind using a fixed volume. Viable cell concentrations are plotted against the time post-infection. Each color indicates a different virus passage.
